# Quantitative Analysis of Decoquinate Residues in Hen Eggs through Derivatization-Gas Chromatography Tandem Mass Spectrometry

**DOI:** 10.3390/foods13010119

**Published:** 2023-12-29

**Authors:** Yali Zhu, Lan Chen, Yawen Guo, Pengfei Gao, Shuyu Liu, Tao Zhang, Genxi Zhang, Kaizhou Xie

**Affiliations:** 1 College of Animal Science and Technology, Yangzhou University, Yangzhou 225009, China; 15152619632@163.com (Y.Z.); dx120200135@yzu.edu.cn (Y.G.); mz120211479@stu.yzu.edu.cn (S.L.); zhangt@yzu.edu.cn (T.Z.); gxzhang@yzu.edu.cn (G.Z.); 2 Joint International Research Laboratory of Agriculture & Agri-Product Safety, Yangzhou University, Yangzhou 225009, China; dz120200026@yzu.edu.cn (L.C.); mz120201383@yzu.edu.cn (P.G.); 3 College of Veterinary Medicine, Yangzhou University, Yangzhou 225009, China

**Keywords:** decoquinate, egg, precolumn derivatization, LLE-SPE, GC-MS/MS

## Abstract

A novel precolumn derivatization-gas chromatography tandem mass spectrometry (GC-MS/MS) method was developed to detect and confirm the presence of decoquinate residues in eggs (whole egg, albumen and yolk). Liquid-liquid extraction (LLE) and solid phase extraction (SPE) were used to extract and purify samples. The derivatization reagents were pyridine and acetic anhydride, and the derivatives were subjected to GC-MS/MS detection. After the experimental conditions were optimized, satisfactory sensitivity was obtained. The limits of detection (LODs) and limits of quantification (LOQs) for the decoquinate in eggs (whole egg, albumen and yolk) were 1.4–2.4 μg/kg and 2.1–4.9 μg/kg, respectively. At four spiked concentration levels, the average recoveries were 74.3–89.8%, the intraday RSDs ranged from 1.22% to 4.78%, and the inter-day RSDs ranged from 1.61% to 7.54%. The feasibility and practicality of the method were confirmed by testing egg samples from a local supermarket.

## 1. Introduction

Coccidiosis is a common infectious disease in layer culture, and the primary pathogens are *Eimeria tenella* in the cecum of chicks and Eimeria poisoning in the small intestine [1]. This disease causes large-scale death of chickens and heavy losses to the global chicken industry [2]. Animal coccidiosis, which has a development history of over 20 years, is often prevented and treated by decoquinate, an antimicrobial agent derived from quinolones [3]. As a prevention and treatment of coccidiosis, decoquinate kills coccidial sporozoites at the sporozoite stage, inhibits spore and growth when cracking colonization bodies are first generated and prevents further harmful development inside animal bodies [4,5,6]. Because of its high efficiency and broad-spectrum characteristics, decoquinate is widely used in the chicken industry and effectively controls Eimeria in pigs, rabbits and ruminants [7,8]. Decoquinate is insoluble in water and was initially added to feed as a premix to prevent coccidiosis [9]. According to new data provided by the E.U. Feed Additives and Products Panel in 2021, the minimum dose range for decoquinate was 30 mg/kg in full-price feeds [10].

Despite its advantages, high doses of decoquinate can cause fetal toxicity and affect fetal skeletal development [11]. If an individual consumes an animal-derived food (such as eggs) that contains decoquinate residue, their health may be negatively affected. Different countries and organizations have formulated maximum residue limits (MRLs) for decoquinate in chicken tissues to promote the international trade of animal food and ensure human food safety. These MRLs are usually based on scientific research and risk assessment. They may vary according to national and regional regulations (those from China and the FDA range from 1000–2000 μg/kg, and those from Japan range from 100–2000 μg/kg) [12,13,14,15,16]. Most of these countries stipulated that decoquinate use is prohibited during lactation and egg production, and no MRL has been specified for eggs. Compared with the MRL standards for decorticates issued by the FDA, the European Union and China, the MRL standards stipulated in Japan are the most stringent. Therefore, this study mainly refers to the MRL standard specified for decoquinate use in Japan (100.0 μg/kg) and uses the relevant requirements of E.U. 2002/657/E.C [17]. To verify the analytical methods, this study established and demonstrated a precolumn derivatization-gas chromatography tandem mass spectrometry (GC-MS/MS) to analyse and detect decoquinate residues in egg samples (whole egg, albumen, and yolk).

At present, decoquinate residues are measured through HPLC-UVD (high-performance-liquid-chromatography-ultraviolet detection) [18], HPLC-DAD (diode array detection) [19], HPLC-FLD (fluorescence detection) [20], LC-MS/MS (liquid-chromatography-tandem mass spectrometry) [21,22,23], and surface-enhanced Raman spectroscopy [24]. One of the most widely used detection methods is LC-MS/MS, but the instruments and equipment are expensive and unsuitable for most laboratories. Based on a literature review, no method has been reported to analyze decoquinate residues in eggs with GC-MS/MS. This study aimed to develop an effective GC-MS/MS method to determine and analyze decoquinate residues in eggs.

## 2. Materials and Methods

### 2.1. Materials

A decoquinate (CAS: 18507-89-6, purity ≥ 98%) standard was obtained from Dr. Ehrenstorfer GmbH (Augsburg, Germany). Chromatographically, pure methanol and acetonitrile were purchased from Merck (Mumbai, India). Ethyl acetate, acetic anhydride, acetic acid, n-hexane and trichloromethane were all provided by Sinopharm Chemical Reagents Co., Ltd. (Shanghai, China). Pyridine and nylon needle filters (organic phase, 0.22 µm) were obtained from Shanghai Macklin Biochemical Technology Co., Ltd., Shanghai, China and Anpel Experimental Technology Co., Ltd., Shanghai, China respectively. An Oasis HLB SPE column (column capacity of 3 mL) was purchased from Waters Company. The ultrapure water used in the experiment was made in the laboratory (UK Kertone Co., Ltd., Daventry, UK).

### 2.2. Standard Solutions

Decoquinate (10.33 mg) was accurately weighed and placed in a 10 mL brown volumetric flask. The standard stock solution (1.0 mg/mL) of decoquinate was prepared by dissolving decoquinate in 4% acetic acid trichloromethane solution with a constant volume. The decoquinate stock solution (1.0 mg/mL) was diluted with trichloromethane to prepare standard working solutions with concentrations of 100 μg/m, 10 μg/m and 1 μg/mL. All solutions were stored in a freezer at −80 °C.

### 2.3. Sample Preparation

The Jinghai Poultry Company (Nantong, China) provided all hen eggs used during the study, and the experimental animals were allowed to feed without the added target compound and drink freely. Chicken whole egg, albumen and yolk were homogenized separately with an automatic grinder (JXAUTO-4L, Shanghai Jingxin Industrial Development Co., Ltd., Shanghai, China) and stored and sealed in a −20 °C refrigerator.

Egg samples (2.0 ± 0.01 g) were accurately measured into 50 mL polypropylene centrifuge tubes, vortexed for 1 min, and left to stand for 10 min. An acetonitrile aqueous solution (80%, 5 mL) was added, vortexed, mixed for 10 min, and then ultrasonically extracted for 10 min. The sample was centrifuged at 5500 r/min for 10 min, and then the upper liquid layer was transferred to a new centrifuge tube. The extraction was repeated once with 5 mL of the extract, and the mixture was centrifuged at 8000 r/min for 10 min. The supernatant was combined and saved, before 10 mL of acetonitrile-saturated hexane was added, vortexed and shaken for 5 min, and left to stand. After the extract was layered, the n-hexane layer was discarded. The extract was dried under nitrogen flow at 50 °C and reconstituted with five millilitres of 30% acetonitrile solution. Sample purification was conducted using an Oasis HLB SPE cartridge. Before sample loading, the cartridge was sequentially preconditioned with 3 mL of methanol followed by 3 mL of water to ensure proper activation and equilibration. The flow-through was discarded after introducing the sample solution to the cartridge. The column was washed with 3 mL of 30% acetonitrile and then with 3 mL of water under vacuum conditions to remove any undesired matrix effects. Lastly, 3 mL of acetonitrile was used to elute the target compounds. The eluent was collected in a 10 mL glass centrifuge tube and dried in a gentle stream of nitrogen at 50 °C.

### 2.4. Sample Derivatization

Acetic anhydride was used as the derivatization reagent. With added pyridine, the acetylation reaction occurred with the decoquinate ester, and the derivative was preliminarily determined to be acetyl decoquinate ester (the equation is shown in Figure S1). The function of pyridine in derivatization is to react with acid and promote a positive reaction. Concentration gradient experiments were then performed with the drug and derivatives, which confirmed that the derivative was acetyl decoquinate ester (Figure S2).

Based on the mass spectra of the derivatives, an ion with *m*/*z* 231.1 was chosen as the precursor ion. After determining the parent ions, the instrument parameters were optimized with automatic selective reaction monitoring (auto SRM). Information on the daughter ions produced from the parent ion under different collision energies was gathered, and two fragment ions with the optimal collision energies and stable abundance ratios were selected to prevent interference from other substances. Each target compound had at least two monitoring ion pairs for adequate characterization and quantification. Finally, ions exhibiting *m/z* 230.1 and *m/z* 229.1 were selected as the daughter ions, and the parent and daughter ions formed monitoring ion pairs. The retention time and relevant mass spectrometric parameters for acetyl decoquinate are shown in Table 1.

Trichloromethane (1 mL) was added to the reconstituted extract and vortexed for 1 min. The mixture was reacted for 3.5 h at 25 °C in the dark after 300 µL pyridine was added and 150 µL acetic anhydride was successively added. After reconstitution, the solution was filtered through a 0.22 µm organic-phase nylon syringe filter, and the filtrate was used for GC-MS/MS.

### 2.5. Instrument Conditions

Using a Micro1300 gas chromatograph equipped with a TSQ 8000 quadrupole tandem mass spectrometer with an autosampler Triplus RSH (Thermo Fisher Scientific Co., Ltd., Waltham, MA, USA), the analyte of interest was screened. A TG-5MS capillary column (30.0 m × 0.25 mm, 0.25 m) was used to separate the samples (Thermo Fisher Scientific Co., Ltd. Waltham, MA, USA). Helium (99.999%, 60 psi) was applied as the carrier gas. The flow rate remained constant at 1.0 mL/min. The conditions for gas chromatography were as follows: the carrier gas was helium; the rate was 1.0 mL/min; and the oven temperature was programmed at 100 °C for 1 min, heated to 220 °C at 100 °C/min and held for 1 min. Then, the temperature was raised to 290 °C at 220 °C/min and remained there for 13 min. The injection temperature was 280 °C, and the solvent delay time was 3 min; splitless injection was used with a shunt time of 1 min. The analyses were run with a carrier gas and constant current mode; the Shunt was 50.0 mL/min; the carrier gas saved 2 min; the carrier gas flow was 20.0 mL/min; and the injection volume was 1.0 μL. The MS/MS conditions were as follows: the ionization mode was electron ionization (EI); the ionization voltage was 70 eV; A collision gas of 99.999% argon at a pressure of 40 psi was used in the collision; the temperature of the ion source and transmission line was 280 °C; the scanning mode was full-scan mode (SCAN) for qualitative results; and response monitoring (auto SRM) was selected for qualitative and quantitative results.

### 2.6. Method Parameters

#### 2.6.1. Linearity

We diluted decoquinate ester’s standard working solution to various concentrations. The final dilution concentration corresponds to the LOQ concentration, 50, 100, 150, 200, and 250 μg/kg in the sample. GC derivatized the diluted standard solution-MS/MS. Six parallel experiments were conducted for each concentration, and peak area calculations were used. A matrix standard curve was established for the quantitative analysis of test samples, with the horizontal axis (*x*) representing the concentration of decoquinate added to three different matrices, and the ordinate (*y*) was the peak area of the quantitative ion pair of acetyl decoquinate.

#### 2.6.2. Matrix Effect

The matrix effect (ME) was assessed using the slope of the standard curve following the establishment of the GC-MS/MS technique. In addition to preparing the matching standard curve for each matrix, the solvent standard curve was also prepared simultaneously. Standard working solutions at concentrations of 6.8, 100, 200, 300, 400 and 500 ng/mL were prepared by gradually diluting the decoquinate standard stock solution with trichloromethane. The standard curves were plotted, and the slope of the standard curves was used to calculate the ME for the whole egg. A series of standard solutions with concentrations of 4.2, 100, 200, 300, 400, and 500 ng/mL were prepared, standard curves were developed, and the ME of egg albumen was calculated. A series of standard solutions with concentrations of 9.8, 100, 200, 300, 400, and 500 ng/mL were developed, the standard curve was developed, and the ME of the egg yolk was calculated. The ME calculation formula was as follows:

$$ME\;\left(\%\right)\;=(\frac{Slope\;matrix-matched\;calibration\;curve}{Slope\;solvent\;standard\;curve}-1)\times 100\%$$

When −20% ≤ ME ≤ 20%, this showed a weak ME. When −50% ≤ ME ≤ −20% or 20% ≤ ME ≤ 50%, there is a medium ME. When ME ≤ −50% or ME > 50%, there is a strong ME.

#### 2.6.3. LOQ and LOD

Separate blank matrix extracts of whole eggs, egg albumens, and egg yolks were prepared, and the standard working solution of decoquinate was diluted with them. The optimized GC-MS/MS technology was used for both detection and analysis. Set 6 parallels for each concentration were used to calculate the average of the signal-to-noise ratio (S/N) for each engagement. The method’s LOD was the concentration of S/N ≥ 3, and the LOQ was the concentration of S/N ≥ 10.

#### 2.6.4. CCα and CCβ

Determination and detection tolerance are important indexes used to evaluate the detection methods of pesticide residues in the E.U. 2002/657/E.C. The CCα is the limit beyond which it is possible to conclude with a probability of error equal to α that a sample is non-compliant. The likelihood that the detection result for the analyte in the sample does not correspond to the prescribed conclusion (false positive) can be determined. For veterinary pharmaceutical products with clear maximum residue limits (MRL), CCβ represents the concentration of the target substance MRL in the sample that can be accurately detected, and the detection probability of the MRL in the sample can be calculated on this basis. For veterinary pharmaceutical products that do not indicate the MRL value, CCβ represents the lowest concentration that can accurately detect the target substance in the sample, and the probability that the negative sample does not meet the requirements (false negative) can be calculated on this basis. The CCα of banned drugs can be calculated using the calibration curve method. A blank sample was randomly selected, and the target substance was added according to the equidistant gradient in an amount equal to or higher than the minimum amount needed by the reaction. After the sample was analyzed, the graph was plotted according to the added level. The corresponding concentration was added to the *Y*-axis intercept (*Y*-axis extrapolation method) plus 1.64 times the standard deviation of the reproducibility in a laboratory, which is CCα (α = 5%); Decoquinate standard was added to 20 blank samples to determine the limit level, the samples were analyzed, and the internal reproducibility standard deviation of the average detected content in the laboratory when CCα was added with 1.64 times the CCα concentration—this was the CCβ (β = 5%).

CCα = LOQ + 2.23 × SD (α = 5%)

CCβ = CCα + 1.64 × SD (β = 5%)

#### 2.6.5. Recovery and Precision

The blank whole egg, egg albumen, and egg yolk samples (2.0 ± 0.01 g) were accurately weighed, and the decoquinate standard working solution was added to the three matrices. The LOQ, 0.5 MRL, 1.0 MRL and 2.0 MRL (*n* = 6) were the concentrations. The optimized sample pretreatment and derivative methods were used to treat the sample, and the optimized GC-MS/MS method was used to examine the final derivative quantitatively. Substitute the peak area into the matrix standard curve to obtain the corresponding concentration and recovery rate of the sample. Intra-day precision: when the same concentration is added at different times on the same day, the same instrument is used for detection and analysis, and the same standard curve is used to calculate the recovery rate. The same operator used the same instrument to analyze six replicate samples with the same spiked concentration on different days of the week and utilised different matrix standard curves to calculate the recovery rate and thereby obtain the inter-day precision.

## 3. Results and Discussion

### 3.1. Optimized Sample Preparation

A derivatization test must be conducted in this experiment before the instrumental method is activated. The derivatisation reaction must be heated and refluxed in the early stage of the derivatization test. Methanol cannot be used as the solvent because the acetic anhydride derivatization reagent undergoes an esterification reaction with alcohol. Therefore, 100% trichloromethane was used to dissolve decoquinate ester, and it was found that the solubility of decoquinate ester in trichloromethane was very low. Thus, the solubility of the drug was increased by adding acid to trichloromethane; after multiple experiments, it was found that a 4% trichloromethane-acetate solution provided the highest decoquinate ester solubility. Ten milligrams of the decoquinate ester standard were fully dissolved in 10 mL of the 4% acetic acid-trichloromethane solution.

In previous reports, ethyl acetate, acetonitrile, and acidified acetonitrile [25,26] were mainly used as extractants. After the samples were purified, good extraction results were obtained. Therefore, this study compared the effects of 4% acetic acid acetonitrile, acetonitrile and acetonitrile ethyl acetate (50%:50%) on the extraction effect of decoquinate in chicken whole egg, albumen and yolk samples. The extraction effect of acetonitrile on the decoquinate in eggs is shown in Table 2. All the above extractants effectively extracted decoquinate from poultry eggs. However, the recoveries with 4% acetonitrile acetate solution and acetonitrile ethyl acetate (50%:50%) solution were unstable, while the recovery rate of decoquinate extracted from eggs with acetonitrile was above 80%, which was noticeably higher than that of the other two solution systems. In addition, extraction of the decoquinate was performed with different concentrations of the acetonitrile aqueous solution, and it was found that an 80% acetonitrile aqueous solution best extracted the egg matrices.

The fat content in eggs is high [27], so fat removal is unavoidable in pretreatment methods [28]. The most widely used method is solvent extraction. The solvent most commonly used in the pretreatment of veterinary drug residues is n-hexane [29]. The purification effects of solvent extraction and low-temperature freezing methods were compared in this study. When the extraction solution was purified with the two methods, the SPE cartridge showed that fat removal was more effective with n-hexane. Finally, acetonitrile-saturated n-hexane (10 mL) was used for fat removal from the egg sample.

SPE is among the main techniques used to enrich target compounds and purify samples. In this study, SPE was selected by considering the properties of decoquinate and the type of SPE cartridge used in prior research [30]. Decoquinate is a weakly polar compound and is more suited for reversed-phase solid-phase extraction than for lipophilic-hydrophilic mixed solid-phase extraction, as the extraction effect of the column was better [31]. Cleanert PEP is a functionalized polystyrene/divinylbenzene SPE cartridge. It exhibits strong absorption for a variety of polar compounds and is suitable for sample purification of different substrates. It has similar characteristics to the Oasis HLB SPE cartridge but is less expensive. This study examined the effects of three SPE cartridges (the Waters Oasis HLB, Strara-X and Cleanert PEP cartridges), and the experimental results were evaluated from the following perspectives: purification effect, recovery and workload. The results showed that Waters Oasis HLB and Cleanert PEP cartridges obtained satisfactory recoveries, the chromatographic peaks were complete, and the target compounds could be completely separated from the interfering components in the sample. When the Strara-X cartridge was used, the final recovery of the cleaned-up samples was 35.37–37.16%. Although the column passing speed of Cleanert PEP was faster than that of HLB, which could shorten the working time, the recovery rate of HLB was higher than that of Cleanert PEP. In our study, a Waters Oasis HLB column was selected for sample purification.

### 3.2. Optimization of the Derivatization Conditions

This study optimised derivatisation conditions to adopt a single-factor experimental design method. One millilitre of the mixed 100.0 μg/mL standard working solution was placed in eight 10 mL glass centrifuge tubes, 100 μL of pyridine was added, and then 50, 100, 150, 200, 250, 300, 350, and 400 μL of acetic anhydride was added. Six replicate experiments were set up, and the reactions were run for 1 h in the dark at 25 °C. After the reaction, the product was concentrated, reconstituted, filtered and analyzed by the optimized GC-MS/MS method. The optimal dose of acetic anhydride needed for derivatization of the decoquinate was 150 μL.

One millilitre of the mixed 100.0 μg/mL standard working solution was placed into six 10 mL glass centrifuge tubes, 150 μL of acetic anhydride was added, and then 150, 300, 450, 600, 750, and 900 μL of pyridine was added. Six replicate experiments were performed at 25 °C for 1 h in a light-proof environment. After the reaction, the product was concentrated, redissolved, filtered, and detected by optimized GC-MS/MS. When 300 µL of pyridine was added, the derivative product had the highest response value and the largest peak area.

The optimal reaction time for the acetic anhydride and pyridine was established after determining the appropriate dosages of acetic anhydride and decoquinate ester. One millilitre of mixed standard working solution (100.0 μg/mL) was added to a 10 mL glass centrifuge tube, and pyridine (300 μL) and acetic anhydride (150 μL) were added. After vortex reaction for 1 min, the test tube was kept in the dark at 25 °C for 0.5, 1, 1.5, 2, 2.5, 3, 3.5, 4, 4.5 and 5.5 h. After the derivatization reaction, the product was concentrated, reconstituted, filtered, and detected by optimized GC-MS/MS. When the derivatization time was from 0.5 h to 5.5 h, the peak area of the derivative increased continuously and reached the maximum at 3.5 h, then decreased slightly and stabilized. The optimal derivatization reaction time was therefore 3.5 h. Finally, the optimal derivatization conditions were identified as 25 °C, 3.5 h, 150 μL of acetic anhydride and 300 µL of pyridine.

### 3.3. GC–MS/MS Optimization

The most commonly used quartz capillary column was used in this study due to the chemical properties of the target substance and the laboratory conditions. Decoquinate is a weakly polar compound transformed into a new acetyl compound by an acetylation reaction. Compared with the original drug, the polarity is further reduced. Therefore, according to the properties of the derivative products, a suitable analytical column was selected for the preliminary test to separate the target in the weakly polar chromatographic column. The nonpolar chromatographic column TG-1MS (30.0 m × 0.25 mm, 0.25 μm) was initially chosen to separate the analyte. After multiple tests, it was determined that the test results were unstable and the target peak could occasionally not be completely separated from the impurity peak in the chromatogram. Moreover, after the column flow rate and temperature program were increased, the peak shape of the chromatographic peak was wider and not sharp. Next, the chromatographic column was replaced with TG-5MS for testing. Multiple tests showed that the derivative products had high response values, the chromatographic peaks were complete, and the target chromatographic peaks showed no tailing. Therefore, a TG-5MS (30.0 m × 0.25 mm, 0.25 μm) column was selected in this experiment to analyze the residues of decoquinate in hen eggs.

Derivatized decoquinate produces a new compound that cannot be temperature programmed based on the boiling point of the original drug. During the research procedure, the acquisition time of each temperature can only be increased to the greatest extent possible to use the derivative products’ retention time to estimate the approximate range of the boiling point. At the beginning of the test, the initial temperature was set to 100 °C and held for 1 min. Then, the temperature was raised at a rate of 30 °C/min to 220 °C and held for 1 min, and, finally, the temperature was increased at a rate of 20 °C/min to 280 °C and held for 16 min. The boiling point of the derivative product was therefore approximately 280 °C. Next, the peak time of the target peak was shortened by changing the temperature-increasing program, and the detection speed was accelerated. The program was then adjusted to an initial temperature of 100 °C, the temperature was maintained for 1 min, heated to 220 °C at a rate of 30 °C/min, held for 1 min, and finally heated to 290 °C at a rate of 30 °C/min and held for 13 min. With this temperature program, the retention time of the chromatographic peak of the target compound was approximately 17.40 min. The heating program was adjusted to an initial temperature of 100 °C, held there for 1 min, heated to 200 °C at a rate of 15 °C/min, held there for 2 min, and finally heated to 280 °C at a rate of 20 °C/min and held there for 7 min; At this time, the chromatographic peak of the derivative product always appears when the second data collection. Therefore, the optimized heating program setting (Section 2.5) is obtained.

If the temperature of the ion source and transmission line is too high or too low, it will directly affect the analysis results of samples and reduce the service life of the ion source. The scanning range and acquisition time are mainly selected according to the chemical properties of the target and the comparison results of many experiments. This experiment is based on the molecular weight of derivative products combined with the mass-to-charge ratio and concentration gradient test results in mass spectrometry. Then, the mass spectrometry conditions are optimized after the derivative products are determined. The TSQ 8000 mass spectrometer automatically tunes the ion source in autotune mode to reach the optimal state. When the ion source reached an optima level, an electron ionization (EI) source was selected and scanned in full-scan scanning mode. The scanning range was *m/z* 50–*m/z* 550. After determining the chemical compound to be measured, auto SRM was used for quantitative scanning. According to the results of qualitative and quantitative scanning, the final mass spectrometry conditions were as follows: the temperature of the ion source and transmission line was set at 280 °C; *m/z* 231.1 > 229.1 and *m/z* 231.1 > 230.1 were selected as qualitative ion pairs; and *m/z* 231.1 > 230.1 as quantitative ion pairs; the optimal collision energies were 48 eV and 56 eV, respectively.

**Figure 1:**
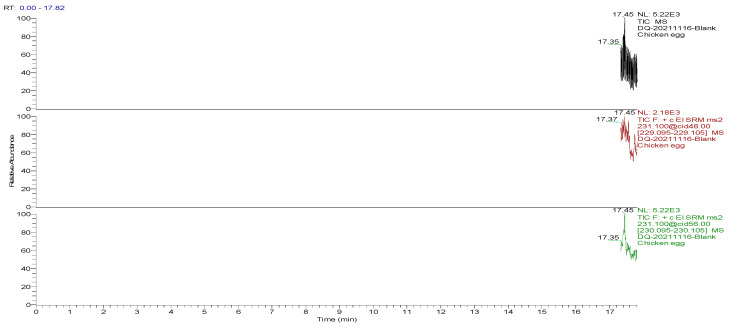
TIC and qualitative and quantitative ion chromatograms for blank hen egg samples.

**Figure 2:**
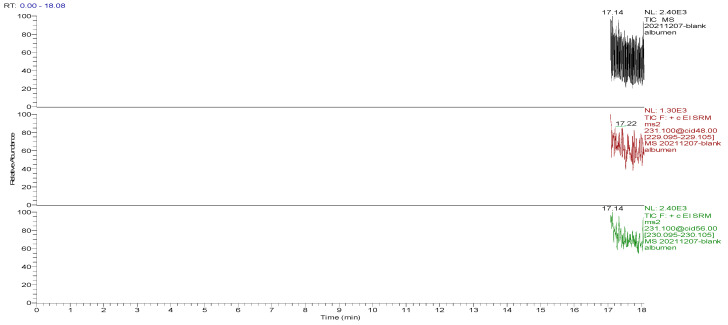
TIC and qualitative and quantitative ion chromatograms for blank albumen.

**Figure 3:**
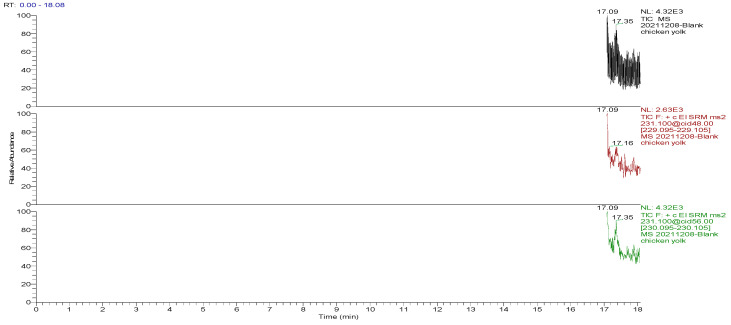
TIC and qualitative and quantitative ion chromatograms for blank yolk.

**Figure 4:**
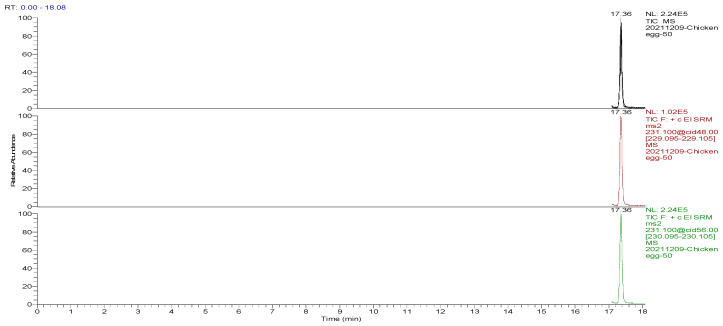
TIC and qualitative and quantitative ion chromatograms for blank hen egg spiked with 100.0 μg/kg decoquinate.

**Figure 5:**
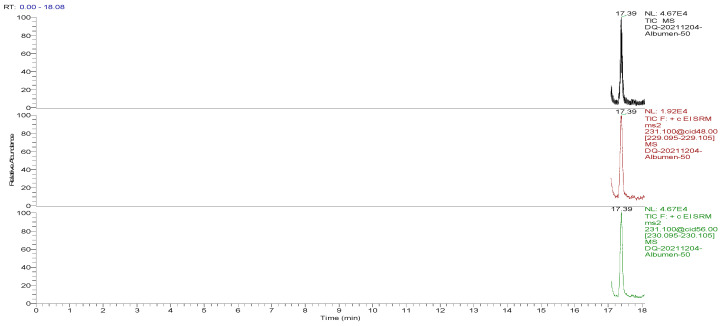
TIC and qualitative and quantitative ion chromatograms for blank albumen spiked with 100.0 μg/kg decoquinate.

**Figure 6:**
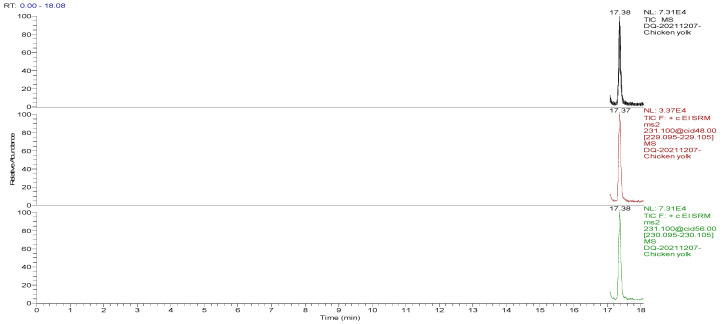
TIC and qualitative and quantitative ion chromatograms of blank yolk spiked with 100.0 μg/kg decoquinate.

The optimized GC-MS/MS method was used to detect the blank and spiked egg samples. The total ion chromatograms (TIC) and the mass chromatograms (MC) of the blank egg samples and the TIC and MC of the standard working solution were supplemented with 100.0 µg/kg decoquinate, as the three blank samples in Figure 1, Figure 2, Figure 3, Figure 4, Figure 5 and Figure 6 show Acetyldecyl quinoxalate showed a high response value, a sharp and non-trailing peak shape and good separation. The retention time of the chromatographic peak of the target compound is approximately 17.40 min.

### 3.4. Quality Parameters

#### 3.4.1. Linearity

In the blank egg samples, the different concentrations of decoquinate were in the range of LOQ-250.0 μg/kg, and the peak area (Y) for the acetyl decoquinate ion pair (*m/z* 231.1 > 230.1) showed an excellent linear relationship with the concentration (X). The linear range, linear regression equation and coefficient of determination of decoquinate in egg samples are shown in Table 3.

#### 3.4.2. Matrix Effect

In this study, the standard stock solution of decoquinate was diluted with chloroform to prepare a standard solution with a concentration of LOQ, 100, 200, 300, 400 and 500 μg/mL, and a standard curve was established to evaluate ME. The MEs of whole eggs, albumen and yolk were calculated, and the results are shown in Table 4. The ME values calculated for these experiments ranged from −13.48% to −7.93%, which corresponded to −20% ≤ ME ≤ 20%. This showed a weak matrix inhibition effect for the whole egg, albumen and yolk samples.

#### 3.4.3. LOD and LOQ

The LODs and LOQs for the samples are shown in Table 3. The LODs of decoquinate in chicken whole eggs, albumen and yolk were 1.8 μg/kg, 1.4 μg/kg and 2.4 μg/kg, respectively, and the LOQs of decoquinate in chicken whole eggs, albumen and yolk were 3.4 μg/kg, 2.1 μg/kg and 4.9 μg/kg, respectively.

#### 3.4.4. CCα and CCβ

Decoquinate is an anti-coccidiosis drug, and its use is prohibited during the egg-laying period. As shown in Table 3, the decision limit of prohibited substances in E.U. 2002/657/E.C. was considered, and the CCα and CCβ were calculated for decoquinate in whole eggs, albumen and yolk at the MRL level.

#### 3.4.5. Recovery and Precision

When the concentration range for decoquinate in blank whole eggs was LOQ–200.0 μg/kg, as shown in Table 5, the recovery rate of decoquinate in blank whole eggs was 74.31–88.53%, the intra-day RSD and inter-day RSD were 2.76–3.32% and 3.16–3.96%, respectively. The recovery of decoquinate in egg albumen was 78.61–89.77%, the intra-day RSD was 1.22–4.78%, and the inter-day RSD was 1.61–7.54%. The spiked recoveries of the four concentrations ranged from 76.08% to 88.13%, the intra-day RSD was 1.59–4.61%, and the inter-day RSD was 1.66–7.38%.

### 3.5. Comparison with Other Analytical Methods

Currently, the primary method for analyzing decoquinate residues in eggs is LC-MS/MS. Our experimental results were compared with those from the liquid chromatography methods established for milk [32] and chicken liver [33], based on the sensitivity, accuracy and experimental conditions—the comparison results are shown in Table 6. Mass spectrometry exhibits the highest sensitivity, but is, compared with HPLC-MS/MS instruments and GC-MS/MS instrumentation, less expensive. Liquid chromatography must be equipped with a liquid mobile phase, and phases can be expensive. Matus et al. [34] detected 17 anticoccidial drug residues, including decoquinate, in chicken by applying HPLC-MS/MS, which capitalized on the high sensitivity inherent to mass spectrometric analysis. However, the sample pretreatment method was unsatisfactory, as the operation steps were complex, and too much time was required. The sample also needs to be vibrated on the horizontal vibrating screen at high speed for 30–45 min. The method established in this research was used to detect the residues of decoquinate in whole eggs, albumen and yolk. This method has, when compared with the current national standard, several advantages, including low detection cost, good stability, satisfactory recoveries and higher sensitivity.

### 3.6. Application with Real Samples

To verify the availability of this method, 40 eggs purchased from a local supermarket were studied with the optimized experimental conditions, and decoquinate was not detected in the tested samples.

## 4. Conclusions

In this study, a precolumn GC-MS/MS confirmatory analytical method was established for the detection of decoquinate residues in eggs. After the method was applied, the LODs for whole egg, albumen and yolk was found to range from 1.4–2.4 μg/kg, the LOQs ranged from 2.1–4.9 μg/kg, andthe recovery rates of the samples were over 74.31%. After the method parameters were optimized, the final method achieved a high recovery rate, low detection cost and good sensitivity, which can provide accurate qualitative and quantitative decoquinate analyses and meet the egg detection requirements. Furthermore, the method was successfully applied to analyze 40 egg samples, and the results demonstrated the reliability of the method. The method provides a novel detection technology to confirm and analyze the presence of decoquinate residues in egg samples.

## Figures and Tables

**Table 1:** Retention time and MS parameters for decoquinate derivatives.

Target Compound	Molecular Weight	Retention Time	Target Compound	Collision Energy (eV)
Acetyl decoxyquine	459.57	17.40	231.1 > 229.1	48
			231.1 > 230.1 *	56

Note: * Quantification ion pair.

**Table 2:** Effects of different extraction reagents on the recovery of decoquinate from whole egg, albumen and yolk (%) (*n* = 6).

Matrix	Acetonitrile	Acetonitrile: Ethyl-Acetate (1:1, *v*/*v*)	4% Acetic Acid Acetonitrile
Whole egg	83.50 ± 0.35	74.88 ± 0.96	82.50 ± 1.02
Albumen	80.23 ± 2.36	77.98 ± 1.19	78.16 ± 2.05
Yolk	86.46 ± 1.73	80.60 ± 0.81	81.18 ± 4.12

**Table 3:** Linear regression equations, determination coefficients, linearity ranges, LODs, LOQs, CCα and CCβ of decoquinate in whole egg, albumen and yolk.

Sample	Linear Regression Equation	Determination Coefficient	Linearity Range (ng/mL)	LOD (µg/kg)	LOQ (µg/kg)	CCα (µg/kg)	CCβ (µg/kg)
Whole egg	y = 603.42x + 15,505	0.9992	3.4–250.0	1.8	3.4	4.7	5.4
Albumen	y = 611.19x + 13,692	0.9993	2.1–250.0	1.4	2.1	2.5	3.3
Yolk	y = 589.88x + 13,850	0.9991	4.9–250.0	2.4	4.9	5.5	6.2

**Table 4:** ME for decoquinate in whole eggs, albumen and yolk.

Sample	Solvent Calibration Curve Equation	Matrix Calibration Curve Equation	ME
Whole egg	y = 677.80x + 58,312, R^2^ = 0.9990	y = 603.42x + 15,505, R^2^ = 0.9992	−10.97
Albumen	y = 663.84x + 55,438, R^2^ = 0.9992	y = 611.19x + 13,692, R^2^ = 0.9993	−7.93
Yolk	y = 681.79x + 68,968, R^2^ = 0.9996	y = 589.88x + 13,850, R^2^ = 0.9991	−13.48

**Table 5:** Recovery and precision for decoquinate were added to blank whole egg, albumen, and yolk (*n* = 6).

Matrix	Added Level (μg/kg)	Recovery (%)	RSD (%)	Intra-Day RSD (%)	Inter-Day RSD (%)
Whole egg	3.4	74.31 ± 1.49	2.01	2.95	3.51
	50.0	82.43 ± 1.78	2.16	3.06	3.68
	100.0 ^α^	86.09 ± 1.97	2.29	2.76	3.16
	200.0	88.53 ± 2.50	2.82	3.32	3.96
Albumen	2.1	78.61 ± 3.07	3.91	4.28	7.54
	50.0	83.62 ± 1.66	1.99	4.78	6.19
	100.0 ^α^	86.98 ± 1.60	1.84	2.05	2.80
	200.0	89.77 ± 0.70	0.78	1.22	1.61
Yolk	4.9	76.08 ± 2.38	3.13	4.61	7.38
	50.0	80.44 ± 1.80	2.24	3.04	4.52
	100.0 ^α^	86.91 ± 1.26	1.45	1.59	1.66
	200.0	88.13 ± 1.44	1.63	3.43	3.99

Note: ^α^ Maximum residue limits.

**Table 6:** Comparison of detection methods for decoquinate.

Matrix	Analytical Method	Chromatographic Conditions	LOD (µg/kg)	LOQ (µg/kg)	Recovery (%)
Milk [31]	UPLC-MS/MS	Agilent Zorbax Eclipse Plus C18	0.78	5.0	≥98.3
Chicken liver [32]	HPLC-UV	Agilent Eclipse XDB-C18	100	200	72.9–96.8
Chicken muscle [33]	HPLC-MS/MS	Agilent Poroshell 120 EC-C18	8	27	74–112
Egg [20]	UPLC-MS/MS	ACQUITY UPLC BEH HILIC	0.004	/	89.9–108.0
The study (Hen egg)	GC-MS/MS	TG-5MS Amine (30.0 m × 0.25 mm, 0.25 μm)	1.4–2.4	2.1–4.9	74.31–89.77

Note: /, Unspecified.

## Data Availability

Data is contained within the article.

## Supplementary Materials

The following supporting information can be downloaded at: https://www.mdpi.com/article/10.3390/foods13010119/s1.
