# Supplementary material for: Quantitative Analysis of Decoquinate Residues in Hen Eggs through Derivatization-Gas Chromatography Tandem Mass Spectrometry
Source: Foods. 2023 Dec 29;13(1):119. doi: 10.3390/foods13010119 (PMC10778401; doi:10.3390/foods13010119)
Supplement: Supplementary file 1 [file foods-13-00119-s001.zip › foods-2766068-supplementary.pdf]

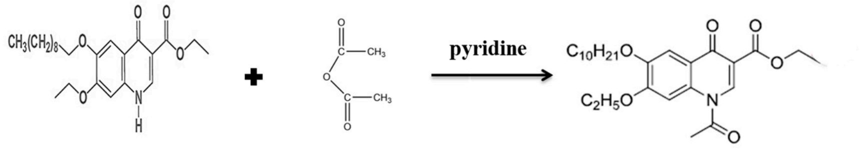

Figure S1. Derivatization of decoquinate with acetic anhydride.

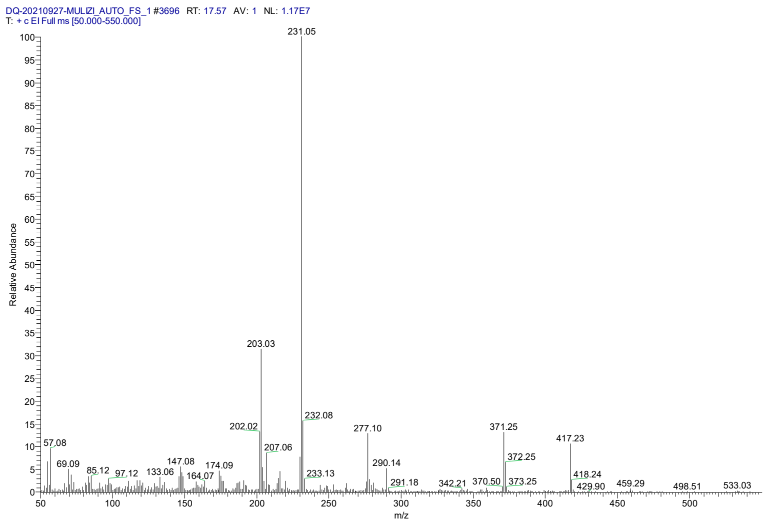

Figure S2. Mass spectra of the decoquinate derivatives.
